# Cognitive flexibility predicts attitudes towards vaccination: evidence from a New Zealand sample

**DOI:** 10.1186/s40359-024-02048-2

**Published:** 2024-10-14

**Authors:** Stephanie Gomes-Ng, Jay K. Wood, Sarah Cowie

**Affiliations:** 1 Department of Psychology, Auckland University of Technology, Auckland, New Zealand; 2 School of Psychology, The University of Auckland, Auckland, New Zealand

**Keywords:** Vaccine hesitancy, Vaccination attitudes, Cognitive flexibility, New Zealand

## Abstract

**Background:**

Vaccine hesitancy (the reluctance or refusal to vaccinate) poses a significant threat to public health worldwide, with declining vaccination coverage resulting in the resurgence of vaccine-preventable diseases (e.g., measles) in recent years. Despite efforts to combat vaccine hesitancy through information-based campaigns and other interventions, vaccine-hesitant attitudes persist. Given that such interventions likely expose individuals to information that conflicts with their own viewpoints about vaccination, *cognitive flexibility* – the ability to adapt one’s thoughts, attitudes, beliefs, or behavior in response to changing information or environmental demands – may play a role in vaccine hesitancy.

**Methods:**

The current study investigated the relationship between cognitive flexibility and attitudes towards vaccination in a sample of New Zealand residents (*N* = 601). Cognitive flexibility was measured using perseverative responses in the Wisconsin Card-Sorting Task, and vaccination attitudes were measured using an adapted version of the Multidimensional Vaccine Hesitancy Scale (MVHS). Linear regression was used with MVHS scores as the dependent variable and cognitive flexibility and sociodemographic variables (age, gender, ethnicity, education level, religion) as predictors.

**Results:**

Cognitive flexibility predicted personal barriers to vaccination (e.g.,” vaccines go against my personal beliefs”), with participants with lower levels of cognitive flexibility reporting greater personal barriers. In contrast, there was no significant relationship between cognitive flexibility and external barriers to vaccination (e.g., “vaccines cost too much”). Additionally, religious participants reported overall higher levels of vaccine hesitancy than non-religious participants.

**Conclusions:**

These findings join others demonstrating that individual differences in cognitive style are associated with attitudes towards vaccination, and tentatively suggest that interventions aiming to reduce vaccine hesitancy may be more effective if combined with techniques to increase cognitive flexibility. To be sure, future work is needed to test the causal relationship between cognitive flexibility and attitudes towards vaccination.

**Supplementary Information:**

The online version contains supplementary material available at 10.1186/s40359-024-02048-2.

## Background

Vaccines are one of the most important achievements in the history of public health: Since their development, vaccines have eradicated several infectious diseases (e.g., smallpox) and decreased morbidity and mortality rates, saving 3.5 to 5 million lives per year [1]. Critically, the extent to which vaccines successfully reduce or eliminate disease transmission depends on vaccination rates in the general population; high rates (> 90%, and even higher for more transmissible diseases) are required to achieve *herd immunity* [2, 3]. Governments and health authorities worldwide aim to increase vaccination rates through immunization programs, public health campaigns, and other policies or interventions. For example, during the recent COVID-19 pandemic, countries mandated vaccination for specific occupations (e.g., healthcare workers) or activities (e.g., concerts), and global campaigns emphasized the importance and safety of the vaccine (e.g., 4).

Despite ongoing efforts to increase vaccine uptake, the number of people who hesitate or refuse to vaccinate themselves or their children is increasing [5]. For example, in Aotearoa^1^ New Zealand (NZ), childhood vaccination rates consistently fall short of the 90–95% target required for herd immunity, dropping to as low as 45% in some demographic groups [6]. Before the COVID-19 pandemic, about 5% of the NZ population expressed anti-vaccination attitudes, while another 25% expressed ambivalence, low confidence, or some concerns with vaccines (7–8). During the pandemic, about 25% of the population were unsure about or would refuse the vaccine [9–11]. These trends are not unique to NZ, and they have led to decreased vaccination coverage and the resurgence of vaccine-preventable diseases, such as measles, in recent years [12–15]. As a result, in 2019, the World Health Organization declared *vaccine hesitancy* to be one of the top ten threats to global health [16].

Individuals may hesitate or decline to vaccinate for a variety of reasons, including (but not limited to) concerns over the safety or efficacy of vaccines, beliefs that vaccines are unnecessary or that disease poses a lower risk than vaccination, difficulties with accessing or affording vaccines, or mistrust in the authorities or health services that promote or deliver vaccines [5, 17–20]. Importantly, attitudes associated with vaccine hesitancy predict behavior; increased vaccine hesitancy is associated with decreased vaccine acceptance (the decision to accept when presented with the opportunity to vaccinate) and vaccine uptake (actual receipt of a vaccine). Thus, identifying the factors that contribute to vaccine-hesitant attitudes is critical for developing effective methods to reduce such attitudes and increase vaccination coverage in the general population [21–24]. Much of the existing research in this area has focused on individual- or demographic-level variables contributing to vaccine hesitancy. For example, vaccine-hesitant attitudes are generally stronger in women (vs. men), ethnic minorities (vs. majorities), rural (vs. urban) communities, more politically conservative or fundamentally religious groups, and in individuals with lower socioeconomic status or education level (e.g., 8–10, 25–31).

## The importance of cognition

In addition to demographic variables, more recently researchers have begun to explore the cognitive factors that underpin attitudes towards vaccination. Making decisions about vaccination is a cognitively effortful process [32, 33]: To illustrate, consider a person faced with conflicting information from different sources about the safety of a new vaccine (as was the case for many people during the pandemic). How does this information strengthen or challenge their pre-existing beliefs about, or experiences with, vaccination? How do they decide which information (if any) to trust? Does exposure to this information alter their attitudes towards vaccination, and in which direction? These kinds of questions are especially pertinent amid the proliferation of information – and *mis*information – brought on by the rise of online media [34–36]. This “infodemic” has made it even more cognitively challenging to make decisions about vaccination, as we must sift through a plethora of competing opinions and information [37, 38].

Moreover, humans often rely on spontaneous, intuitive “quick thinking” when processing information (i.e., ‘System 1’ thinking; 39–41). Such information processing is vulnerable to cognitive biases, such as a bias to favor information that is consistent with, and to reject information that contradicts, one’s existing beliefs (*motivated reasoning*). Indeed, motivated reasoning has been shown to reduce people’s receptiveness to information countering misperceptions about vaccines, food safety, and climate change (e.g., 42, 43). Similarly, when faced with evidence that contradicts one’s beliefs about vaccination, people may incorrectly interpret such evidence to be consistent with their beliefs (*confirmation bias;*^44^). In contrast to such System 1 processing, more conscious, deliberative, and analytical thinking (‘System 2’ processing) is associated with greater willingness to accept, and quicker uptake of, vaccines [45, 46]. Along similar lines, higher scores on tests of general cognitive ability or intelligence [47, 48] and greater ability to estimate event likelihoods accurately [49] are also associated with more positive attitudes towards vaccination. Taken together, these findings suggest that higher cognitive functioning and engagement in more deliberative or analytical information-processing styles predicts more positive attitudes towards, and greater acceptance of, vaccines. The current study adds to this growing body of evidence by investigating the relationship between *cognitive flexibility* and attitudes towards vaccination.

### Cognitive flexibility

Cognitive flexibility is a core component of executive functioning and plays a role in higher-order effortful processes such as reasoning, problem solving, decision making, and planning [50]. Broadly speaking, cognitive flexibility is the ability to adapt one’s thoughts, perspectives, attitudes, or behaviors in response to changes in task or environmental demands. In the context of vaccination, cognitive flexibility may play a role in the development, strength, or persistence of vaccine-hesitant attitudes. For example, when faced with information about vaccination, individuals low in cognitive flexibility may be more likely to rely on spontaneous processing (and hence be vulnerable to cognitive biases) than to engage in deliberative or analytical processing. Indeed, prior work has shown that individuals low in cognitive flexibility are more susceptible to fake news [51], have more extremist views [52–54], are less likely to adjust political beliefs when faced with credible information indicating that their beliefs are incorrect [55], and are less likely to engage in health-protective or pro-environmental behaviors (56–57). These findings suggest that low cognitive flexibility may predict greater vaccine hesitancy.

In further support of this line of reasoning, a handful of studies has shown that executive functioning predicts attitudes towards vaccination. Acar-Burkay and Cristian [32] found that weaker executive functioning predicted less positive attitudes towards vaccines, lower willingness to get the COVID-19 vaccine, and lower trust in health authorities. Similarly, people who discount the value of delayed rewards more steeply in temporal discounting tasks (a pattern reflecting greater impulsivity and weaker executive functioning) express greater vaccine hesitancy and are less likely to have received the COVID-19 vaccine [58, 59]. Other findings show that intellectual humility – the awareness of one’s intellectual fallibility and openness to changing one’s attitudes or beliefs – is correlated with vaccination attitudes, with lower intellectual humility predicting stronger anti-vaccination attitudes [60–63]. While these studies provide strong support for a link between cognitive flexibility and vaccine hesitancy, none involve direct behavioral measures of cognitive flexibility. As a result, whether performance in tasks reflecting cognitive flexibility predicts attitudes towards vaccination remains to be investigated.

## The current study

The primary aim of the current study was to examine the relationship between cognitive flexibility and attitudes towards vaccination. We measured cognitive flexibility using a shortened version of the Wisconsin Card-Sorting Task, a set-shifting task in which the rule used to match cards changes abruptly [64, 65]. Participants must adjust their behavior following each rule change, and the extent to which they perseverate on the previously correct rule provides a behavioral measure of cognitive flexibility. We measured attitudes towards vaccination using an adapted version of the Multidimensional Vaccine Hesitancy Scale (MVHS; 18), which covers a range of factors that may contribute to vaccine hesitancy, such as health risks, costs, accessibility, and physical pain. In line with previous findings demonstrating that higher cognitive functioning is associated with more positive attitudes towards vaccination, we hypothesized that participants with greater cognitive flexibility in the set-shifting task would report lower levels of vaccine hesitancy in the MVHS.

## Methods

All study procedures were approved by The University of Auckland Human Participants Ethics Committee (reference 23822).

## Participants

Data were collected between June 2022 and July 2023. At the start of data collection, the COVID-19 vaccine had been available to the NZ public for about a year [66].

New Zealand residents (*N* = 601; 245 males, 343 females, 7 gender-diverse, and 6 declined to provide gender) aged 18 or over (*M* = 32.93 years, *SD* = 11.58) were recruited via posts on social media (local community groups on Facebook and Reddit; *N* = 117) and on the online data-collection tool Prolific (*N* = 484) [67]. The sample size was based on resource constraints and a one-year timeline for recruitment.

Posts on social media invited potential participants to click a link to learn more about the study, and then they were given a unique link to participate. After participating, participants recruited via social media submitted their contact details on a separate form, and received a $40 NZD (NZ dollars) gift voucher. Participants recruited on Prolific were compensated at a rate of $26.47 NZD per hour. All participants passed all attention checks during the experiment, so no data were excluded from analyses. There were eight attention checks, randomly interspersed among trials in the Card-Sorting Task and among items in the MVHS. These asked participants to choose a specific stimulus (e.g., “select the right-most card”) or response option (e.g., “select Agree for this item”).

44% of participants were located in the Auckland region, followed by Canterbury (15.1%), Wellington (14.5%), Waikato (5.8%), and Otago (5.2%). Most participants identified as NZ European (68.6%), followed by Asian (30.3%), indigenous Māori (8.8%), Pacific peoples (4%), and other ethnicities (4.2%). Almost all participants had at least one educational qualification (high school: 15.1%, tertiary certificate or diploma: 16.1%, bachelor’s degree: 41.1%, postgraduate degree: 23.4%), and most participants identified as non-religious (59.2%) followed by Christian (29.2%).

## Procedure

The study ran on Psytoolkit [68, 69]. Participants completed a Card-Sorting Task and the Multidimensional Vaccine Hesitancy Scale.

### Card-sorting Task (CST)

The card-sorting task was a shortened version of the Wisconsin Card-Sorting Task [64, 65]. Participants were informed that they would be playing a “card matching game” and were given the following instructions before beginning the task: *“In the first part of this study*,* you’re going to play a matching game. Your task is to figure out the “rules” for matching cards together. You’ll see a card at the bottom of the screen*,* and a set of four cards to choose from. One of the cards in the set matches the card at the bottom of the screen. Choose the card that you think matches by clicking it with your mouse.”*

**Fig. 1:**
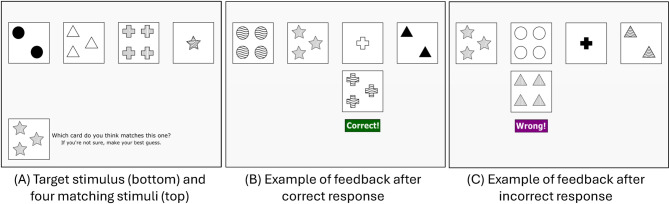
Card-sorting task. Panel A shows the start of a trial, with the target stimulus in the bottom-left corner and the four choice stimuli in a row at the top of the screen. Panel B shows an example of feedback after a correct response matching the Shape dimension. Panel C shows an example of feedback after an incorrect response

Figure 1 shows a typical trial. Participants were presented with a target stimulus card at the bottom of the screen, and chose a matching card from the row of four cards presented at the top of the screen. Each card consisted of three dimensions: *Number* (1 to 4), *Shape* (Circle, Cross, Star, Triangle), and *Pattern* (Solid Filled, Solid Empty, Horizontal Waves, Vertical Lines). The cards for each trial were chosen randomly. To remind participants of the task, a prompt was displayed at the bottom of the screen which stated, “Which card do you think matches this one? If you’re not sure, make your best guess.”. There were six phases, and the correct matching criterion in each phase was, in order: Shape, Number, Pattern, Shape, Number, Pattern. Correct responses (i.e., responses matching the criterion in the current phase) were followed by a sound effect (“ding!”) and the word “Correct!” appeared on the screen for 1.2 s. Incorrect responses were followed by a different sound (a “wrong buzzer”) and the word “Wrong!” appeared on the screen for 1.2 s. Each phase lasted for 12 trials or until 10 consecutive correct responses, whichever occurred first.

## Multidimensional Vaccine Hesitancy Scale (MVHS)

Participants completed the MVHS [18], which consists of subscales that measure different factors that may contribute to vaccine hesitancy. Each subscale consists of four items each, and participants rate their agreement with each item on a scale from 1 (strongly disagree) to 7 (strongly agree). Lower scores on the MVHS indicate lower levels of vaccine hesitancy. We included the eight subscales from the standard MVHS (Health Risks, Cost, Physical Pain, Inconvenience, Personal Reactions, Access, Healthy, and Forget), and two additional subscales from the extended version (Distrust and Beliefs). Table 1 lists the ten MVHS subscales included in the current study, with an example item from each subscale.

**Table 1:** MVHS subscales included in the current study

Subscale	Example Item	Cronbach’s α
Health Risks	Vaccines can cause long-term issues	0.83
Cost	Vaccines cost too much	0.88
Physical Pain	I worry about needles when getting a vaccine	0.90
Inconvenience	I do not have the time to get a vaccine	0.87
Personal Reactions	I have allergic reactions to most vaccines	0.82
Access	Vaccines are unavailable where I live	0.80
Healthy	I do not need vaccines because I rarely get sick	0.91
Forget	Getting vaccines often slips my memory	0.86
Distrust*	I do not trust the creators of vaccines	0.89
Beliefs*	Vaccines go against my beliefs	0.92

Note * indicates subscale from the extended MVHS

## Community influences

To explore the influence of the community on attitudes towards vaccination, we asked participants to rate their agreement, on a scale from 1 (strongly disagree) to 7 (strongly agree), with the following four items: [1] I do not need vaccines to keep my whānau^2^ and community safe; [2] Being vaccinated is important for the health of others in my whānau and community; [3] I will get vaccinated if my family wants me to; and [4] My close friends’ and family’s beliefs about vaccines are important to me. Items 2 to 4 were reverse coded.

### Demographic questions

Participants reported their age, gender, ethnicity, highest level of education, religious affiliation, and region of residence. Participants entered their age in years, and responded to the other questions by choosing from a list of options which were drawn from the NZ census (see Supplementary Materials). Participants could decline to answer any demographic question.

### Data analysis

#### Validation of the MVHS factor structure

The present study is the first to use the MVHS in Aotearoa NZ. Therefore, we assessed its applicability to an NZ sample using confirmatory factor analysis (CFA), and tested the original factor structure proposed by Howard [18]. We conducted a second-order CFA using the 32 items from the standard MVHS. The four items from each subscale loaded onto their respective first-order latent factor, and each first-order latent factor loaded onto one second-order latent factor reflecting ‘vaccine hesitancy’. As in Howard’s original model, we allowed pairs of items within subscales with strong modification indices (> 30) to covary. Model fit was assessed using the comparative fit index (CFI), incremental fit index (IFI), root mean squared error of approximation (RMSEA), and χ²/df. Values above 0.92 for CFI and IFI, below 0.08 for RMSEA, and χ²/df < 4 are considered acceptable, while values above 0.95 for CFI and IFI, below 0.05 for RMSEA, and χ²/df < 3 are considered excellent.

### Cognitive flexibility

To measure cognitive flexibility, we programmed a Python 3.8 script to analyze the number of perseverative responses in each phase of the CST using Heaton et al.’s method [70]. Briefly, this method involves defining a perseverative ‘rule’, which is either set to (1) the matching criterion from the previous phase (e.g., at the end of the Shape phase, the rule is set to Shape) or (2) an incorrect dimension if participants make three unambiguous responses in a row matching that dimension (e.g., if participants are in the Pattern phase, but they make three incorrect unambiguous Shape responses, the rule will change to Shape). Responses that unambiguously match the target card according to the perseverative rule (e.g., they only match the target on the Shape dimension) are counted as perseverative. Any responses which match the target card on more than one dimension (e.g., Shape and Number) are also counted as perseverative if they are sandwiched between unambiguous perseverative responses. A greater number of perseverative responses reflects lower cognitive flexibility.

### Main analyses

Our aim was to investigate the relationship between cognitive flexibility and vaccine hesitancy. Specifically, we hypothesized that lower cognitive flexibility would predict higher vaccine hesitancy scores. To test our hypothesis, we ran linear regressions with vaccine hesitancy subscale scores as the dependent variable, and perseverative response rates and demographic variables (age, gender, ethnicity, religion, education level) as predictors. Gender, ethnicity, and religion were dummy-coded with male, European, and non-religious as reference categories, respectively. Eighty-one participants declined to answer one or more demographic questions; these participants were excluded from the regression. Variance inflation factor (VIF) values ranged from 1.00 to 1.14, indicating that multicollinearity between variables was low.

## Results

### Preliminary analyses

#### Validation of MVHS factor structure

The results of the CFA [χ²(451) = 1854.14, *p* < .001] indicated that the model fell short of the cutoffs for acceptable fit, with a CFI of 0.89, IFI of 0.89, RMSEA of 0.08, and χ²/df = 4.11. Inspection of the individual item loadings indicated that while some items loaded strongly onto their first-order latent factor (e.g., Healthy subscale, with loadings ranging from 0.79 to 0.90), factor loadings were weaker for other items (e.g., 0.57 for “I have a medical condition that prevents me from getting vaccines” from the Personal Reactions subscale). Full results of the CFA are reported in the supplementary materials.

Because the CFA fit indices suggested that the model fit fell short of acceptable, and also because we included eight additional items from the extended MVHS and four novel items measuring community influences, we next performed an exploratory factor analysis (EFA) on all 44 items. Kaiser-Meyer-Olkin (KMO = 0.93) and Bartlett’s tests (χ²(946) = 19396.1, *p* < .001) indicated that data were suitable for EFA. We determined the number of factors to extract using the Kaiser criterion and visual analysis of the scree plot, which suggested a two-factor solution. Hence, we conducted an EFA using principal axis factoring with direct oblimin rotation to extract two factors. These factors accounted for 43.8% of the variance, with further factors explaining relatively little additional variance.

Table 2 shows the results of the EFA. One item (“My close friends’ and family’s beliefs about vaccines are important to me”) loaded less than 0.3 onto both factors, and hence was not included in subsequent analyses. All other items loaded more than 0.35 onto one factor with minimal cross-loadings. Examination of the items loading onto each factor suggested that Factor 1 (Cronbach’s α = 0.94) reflected *personal* factors contributing to vaccine hesitancy, such as moral beliefs, distrust in vaccines, and perceptions of one’s health. In contrast, Factor 2 (α = 0.90) reflected *external* barriers to vaccination, including cost, accessibility, and physical pain. Henceforth, we term these factors “Personal” and “External”.

**Table 2:** Results of exploratory factor analysis

Item	Factor 1	Factor 2
Vaccines go against my personal beliefs	**0.86**	− 0.06
Vaccines go against my beliefs	**0.82**	0.02
Vaccines are unsafe	**0.82**	0.05
People in my physical condition do not need vaccines	**0.80**	0.09
Vaccines are used for corrupt purposes	**0.79**	0.02
Vaccines go against my moral beliefs	**0.79**	0.06
I do not need vaccines to keep my whānau and community safe	**0.78**	0.08
My strong immune system eliminates any need for vaccines	**0.75**	0.15
People are unknowing test subjects for vaccines	**0.72**	0.05
I do not trust the creators of vaccines	**0.71**	0.11
I do not need vaccines because I am a low-risk person	**0.71**	0.14
There is not enough testing on vaccines	**0.66**	0.18
I do not need vaccines because I rarely get sick	**0.62**	0.20
Vaccines go against my religious beliefs	**0.62**	0.07
Vaccines can cause certain disorders	**0.60**	− 0.06
I will get vaccinated if my family wants me to*	**0.59**	− 0.06
I have allergic reactions to most vaccines	**0.51**	0.20
Vaccines can cause illness	**0.50**	0.04
Vaccines can cause long-term health issues	**0.50**	− 0.03
I am allergic to certain ingredients in vaccines	**0.42**	0.23
Being vaccinated is important for the health of others in my whānau and community*	**0.41**	− 0.26
I am a high-risk person for having a negative reaction to vaccines	**0.40**	0.16
I have a medical condition that prevents me from getting vaccines	**0.35**	0.09
My close friends’ and family’s beliefs about vaccines are important to me*	0.18	− 0.10
I do not have the time to get a vaccine	0.12	**0.70**
I just never get around to getting vaccines	0.05	**0.70**
I am too busy to get a vaccine	0.19	**0.62**
Vaccines are too expensive	− 0.01	**0.60**
Getting vaccines often slips my memory	0.07	**0.58**
Vaccines cost too much.	− 0.03	**0.58**
I worry about needles when getting a vaccine	− 0.06	**0.58**
I am unable to get vaccines because they cost too much	0.10	**0.58**
Needles bother me when receiving a vaccine	− 0.09	**0.57**
Getting a vaccine is too much of a hassle	0.27	**0.55**
It is difficult to get a vaccine where I live	0.09	**0.53**
I just forget about getting vaccines	0.14	**0.53**
I have a phobia of needles when receiving a vaccine	− 0.05	**0.53**
Without health insurance, vaccines cost too much	− 0.01	**0.52**
Getting a vaccine hurts	− 0.09	**0.48**
There is nowhere to get a vaccine	0.17	**0.44**
Vaccines are unavailable where I live	0.11	**0.36**
Eigenvalue	14.24	5.01
% Variance	32.37	11.39

Note * indicates reverse-coded item

### Cognitive flexibility

In line with previous research showing decreases in cognitive flexibility with age [71], there was a weak positive correlation between perseverative response rates and age, *r*(547) = 0.15, *p* < .001. There were no significant correlations between perseverative response rates and gender (0 = male, 1 = female, other responses were excluded due to small sample size) or education level (ordinal scale). An ANOVA with ethnicity and religion as between-subjects factors indicated significant main effects of both ethnicity [*F*(4, 561) = 3.25, *p* = .012] and religion [*F*(2, 561) = 3.62, *p* = .027], and an ethnicity x religion interaction [*F*(8, 561) = 2.93, *p* = .003]. Overall, religious participants had higher perseverative response rates than non-religious participants, and this difference was particularly apparent for Asians and Europeans (*p* < .005).

**Fig. 2:**
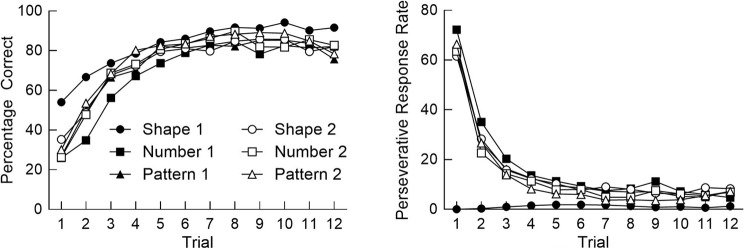
Mean performance across trials in each phase of the Card-Sorting Task. The left panel shows percentage correct, and the right panel shows perseverative response rate. Phases ran in the following order: Shape 1, Number 1, Pattern 1, Shape 2, Number 2, Pattern 2

Figure 2 shows mean percentage correct and the mean rate of perseverative responses (number of perseverative responses divided by total trials) across trials in each phase of the CST. Percentage correct increased and perseverative responses decreased across each phase, indicating that participants learned the new matching criterion in each phase. There were few differences in performance between phases, except for the first phase of the session (Shape), in which few perseverative responses were made because there was no previous criterion upon which to perseverate. These patterns are similar to findings from other behavioral studies that have employed similar reversal-learning tasks, in which accuracy gradually increases and error rates decrease after an abrupt change in contingencies (e.g., 72, 73).

### Main analyses: does cognitive flexibility predict vaccine hesitancy?

Here, we report the results of our main analyses using the two-factor solution suggested by our EFA (Table 2); the results of analyses using Howard’s [18] original factor structure are reported in the footnotes and supplementary materials (the results of both analyses largely aligned).

Overall, participants reported low levels of vaccine hesitancy, with mean scores close to the scale anchor “disagree” for both the External (*M* = 2.16, *SD* = 0.86) and Personal (*M* = 2.08, *SD* = 0.92) subscales. Thus, on average, participants perceived few barriers to vaccination. There were small differences between subscale scores, with higher scores on the External subscale than on the Personal subscale, *t*(600) = 2.07, *p* = .039. There was little relationship between External scores and perseverative response rates (*M* = 0.13, *SD* = 0.07), *r*(599) = -0.01, *p* = .768, whereas there was a weak positive correlation between perseverative responses and Personal scores, *r*(599) = 0.12, *p* = .002^3^. Because regression analysis only included participants who answered all demographic questions (*N* = 520), we also conducted these bivariate correlations after excluding participants with missing demographic data. The results aligned with the bivariate correlations reported for the full sample [External: *r*(519) = -0.01, *p* = .458; Personal: *r*(519) = 0.12, *p* = .003].

Table 3 shows the results of linear regressions with External and Personal scores as the dependent variable, and cognitive flexibility and sociodemographic variables as predictors. Overall, different variables predicted External and Personal factors contributing to vaccine hesitancy. For the External subscale [*F*(10, 509) = 4.11, *p* < .001, *R*^2^ = 0.08], cognitive flexibility, as indexed by perseverative response rates, did not predict scores, whereas age, education level, and religious affiliation were significant predictors with older, more educated, and non-religious participants reporting less external barriers (i.e., lower External scores) to vaccination.

**Table 3:** Regression analysis predicting external and personal vaccine hesitancy factors from cognitive flexibility and demographic variable

	External				Personal		
Predictor	β	t	p		β	t	p
Perseverative responses	0.01	0.20	0.842		0.09	1.99	0.047*
Age	-0.21	-4.65	< 0.001***		0.04	0.78	0.438
Gender (ref: male)	-0.03	-0.77	0.441		-0.08	-1.75	0.081
Ethnicity (ref: European)							
*Māori*	0.02	0.51	0.612		0.05	1.11	0.267
*Pacific peoples*	-0.08	-1.85	0.065		0.03	0.80	0.426
*Asian*	-0.03	-0.64	0.522		-0.10	-2.07	0.039*
*Other*	-0.03	-0.59	0.559		-0.06	-1.29	0.199
Education level	-0.11	-2.45	0.015*		-0.08	-1.73	0.085
Religion (ref: no religion)							
*Christian*	0.10	2.02	0.044*		0.22	4.68	< 0.001***
*Other religion*	0.09	2.07	0.039*		0.11	2.55	0.011*

* *p* < .05, *** *p* < .001

In contrast to External scores, perseverative response rates predicted higher scores on the Personal subscale [*F*(10, 509) = 4.91, *p* < .001, *R*^2^ = 0.09]. Thus, lower cognitive flexibility (i.e., higher perseverative response rates) was associated with higher Personal scores, indicating greater vaccine hesitancy. Additionally, ethnicity and religious affiliation significantly predicted Personal scores; Asian (vs. European) and non-religious participants reported fewer personal barriers to vaccination.

In summary, these findings provide partial support for our hypothesis that lower cognitive flexibility predicts greater vaccine hesitancy: Whereas this was the case for personal factors contributing to vaccine hesitancy (e.g., personal beliefs, perceptions of one’s health), cognitive flexibility did not predict external factors contributing to vaccine hesitancy (e.g., physical pain, cost, accessibility). Instead, the most consistent predictor of both external and personal factors was religious affiliation. In addition to religion, age and education level predicted external barriers, and ethnicity predicted personal barriers (Table 3)^4^.

## Discussion

Despite ongoing efforts to combat vaccine-hesitant or anti-vaccination sentiments and to increase vaccination coverage, vaccine hesitancy continues to pose a threat to public health worldwide [16]. Therefore, elucidating the variables that contribute to vaccine-hesitant attitudes is critical, as such research will help to inform the development of targeted interventions to reduce vaccine hesitancy. To that end, in the present study, we asked whether cognitive flexibility predicts attitudes towards vaccination in a sample of New Zealand residents. In line with our hypothesis, participants with lower cognitive flexibility in a card-sorting task reported greater personal barriers to vaccination (e.g., “vaccines go against my beliefs”). However, no such relationship between cognitive flexibility and external barriers (e.g., “getting a vaccine is too much of a hassle”) was evident. These findings highlight the multidimensional nature of vaccine hesitancy, and suggest that different mechanisms may underlie personal and external factors influencing vaccine-hesitant attitudes.

### The multidimensional nature of vaccine hesitancy

The original Multidimensional Vaccine Hesitancy Scale (MVHS; 18) includes eight factors reflecting different facets of vaccine hesitancy (Health Risks, Cost, Physical Pain, Inconvenience, Personal Reactions, Access, Healthy, Forget; see Table 1). The results of our confirmatory factor analysis did not support this original eight-factor structure, and a follow-up exploratory factor analysis suggested a two-factor structure instead, with some items reflecting ‘personal’ barriers to vaccination and other items reflecting ‘external’ barriers. This separability of personal and external barriers is consistent with other research showing that vaccination coverage (e.g., 74, 75) and engagement in other health-promoting behaviors and health services (e.g., 76–78) depends on both internal and external barriers.

The MVHS is a relatively new scale, and we are the first to use it in the NZ context. Thus, the reasons for why our data did not support the original structure are unclear. One possibility is that the broader context in which we collected data – during the public roll-out of the COVID-19 vaccine in NZ – may have impacted participants’ responses. Specifically, the NZ government’s efforts to increase (and advertise) the accessibility, importance, and safety of the COVID-19 vaccine may have influenced participants to respond similarly to items from different subscales (e.g., reporting few cost-related barriers, low health risks, and low likelihood of forgetting to get a vaccine). This would have resulted in low variability between items from different subscales, and may explain why our exploratory factor analysis indicated that items from different subscales should be collapsed into two broad factors. To be sure, future work should administer the MVHS in NZ with a larger sample and at a time farther removed from the COVID-19 context in order to validate the original eight-factor (versus our two-factor) solution. Sampling from other countries would also provide insight into the extent to which local variables (e.g., government policy, public healthcare access) may influence attitudes towards vaccination on the MVHS.

#### Cognitive flexibility predicts vaccine hesitancy

The present study joins a growing body of research examining the cognitive mechanisms underlying attitudes towards vaccination. Previous studies have shown that more positive attitudes towards vaccines are related to higher performance in tests of cognitive abilities (e.g., Cognitive Reflection Task, intelligence tests), greater executive functioning in the Stroop or temporal-discounting tasks, and higher levels of intellectual humility [32, 58–63]. Additionally, a related body of evidence demonstrates that cognitive flexibility contributes to beliefs in fake news, willingness to adjust political beliefs, and engagement in health-protective or pro-environmental behaviors [51, 53–57]. The current study corroborates these findings, and also extends them: In contrast to previous research, which measured vaccine hesitancy as a single factor, we examined the relationship between cognitive flexibility and *separate* dimensions of vaccine hesitancy. The differential strength of the correlations between cognitive flexibility and different dimensions of vaccine hesitancy suggests that cognition may be more strongly implicated in some aspects of vaccination attitudes than others. Future work should probe the associations between cognitive mechanisms and different dimensions of vaccine hesitancy more closely to gain a clearer picture of the role of cognition in vaccination attitudes.

Our finding that cognitive flexibility predicted personal barriers to vaccination is unsurprising, given that the personal dimension reflects one’s own moral beliefs, trust in authorities, and beliefs about one’s personal health and the safety or efficacy of vaccines (Table 2). These personal beliefs and attitudes – which may or may not be rooted in reality – are likely to be shaped by individual differences in cognition, such as in information processing, analytic thinking, or cognitive flexibility [32, 45, 79]. In contrast, external factors – such as access to clinics, financial costs, or the ‘busyness’ of life – are largely outside of one’s own control; as a result, these factors may not depend, or may depend to a lesser extent, on individual differences in cognition.

While external barriers are probably easier to identify and may lend themselves relatively well to intervention (e.g., reducing costs, providing mobile vaccination services), overcoming personal barriers is considerably more challenging (e.g., 76). Accumulating evidence suggests that despite their widespread usage, interventions that provide information or education on the safety, efficacy, or necessity of vaccination often do not succeed at reducing vaccine-hesitant attitudes or increasing vaccine uptake [33, 80–85]; in some cases, such campaigns may even strengthen anti-vaccination attitudes [33, 85]. This may reflect a tendency towards spontaneous ‘System 1’ processing, which is susceptible to biases that may lead individuals to favor or misinterpret evidence in ways that reflect or strengthen their own pre-existing attitudes [42–44]. Additionally, our findings tentatively suggest that low cognitive flexibility also plays a role, perhaps by making it more difficult for individuals to adapt to new information or to engage in deliberative information processing. In support of this, inducing a “flexibility mindset” that increases deliberative and analytical thinking (e.g., by instructing participants to engage in counterfactual “What if?” thinking) can attenuate cognitive biases and decrease prejudiced attitudes [86]. Relatedly, paradoxical leading-questions interventions, which present individuals with exaggerated or extreme attitude-consistent information, appear to increase cognitive flexibility, leading people to change their anti-refugee beliefs [87]. Thus, the emerging picture here is that cognitive flexibility is an important determinant of attitudes and beliefs in a range of domains, and that it may moderate the effects of interventions that aim to change attitudes or beliefs. This implies that interventions aiming to reduce vaccine hesitancy should consider framing information in ways that encourage deliberative, flexible thinking (see also 79, 88, 89).

One important caveat is that the present results are correlational in nature, and thus whether cognitive flexibility is *causally* related to vaccine hesitancy is an open question for future research. It makes reasonable sense to suggest that low cognitive flexibility leads to the development or persistence of vaccine-hesitant attitudes, but the strength of any such causal relationship, and whether other variables mediate or moderate the relationship, is presently unknown. One way to test this causal relationship in a future experiment is to include manipulations that increase cognitive flexibility. For example, paradoxical leading questions, mindfulness practice, concentrative meditation, or physical exercise have all been shown to increase cognitive flexibility (87, 90, 91). If such techniques are shown to increase cognitive flexibility and, concomitantly to weaken vaccine hesitancy, this would provide some support for a causal relationship between cognitive flexibility and vaccine hesitancy, and would lend support to our suggestion that interventions targeting cognitive flexibility may help to change vaccine-hesitant attitudes.

### The role of sociodemographic variables

Several sociodemographic variables also predicted different dimensions of vaccine hesitancy in the current study. Asian (vs. European) and non-religious participants reported fewer personal barriers, whereas older, more educated, and non-religious participants reported fewer external barriers to vaccination. These patterns are largely consistent with pre-pandemic research conducted in Aotearoa NZ. Asian New Zealanders tend to have high rates of vaccination coverage and more positive attitudes towards vaccination than other ethnic groups [92], while older and more educated individuals may be more aware of, or find it easier to access, healthcare services and credible information about vaccines [8]. Furthermore, given that we collected data during the public roll-out of the COVID-19 vaccine, older participants may have been less likely to report external barriers because they were one of the first groups prioritized to receive the vaccine.

Religious affiliation was the most consistent predictor of, and had the largest effect on, vaccine hesitancy, with religious participants reporting both greater personal and external barriers to vaccination. This is consistent with a large body of international evidence showing that stronger religiosity or spirituality predicts more negative attitudes towards vaccines and lower willingness to vaccinate [93–98]. The underlying reasons for greater vaccine hesitancy in religious individuals are complex, and may reflect theological grounding, concerns over safety or efficacy, low acceptance of or faith in science, moral or social acceptability, or the influence of religious leaders who may explicitly or implicitly oppose vaccination (99–100). While the current study was not focused on religiosity per se, our findings do suggest that religion shapes attitudes towards vaccination independently of cognitive flexibility. As such, further work exploring other cognitive mechanisms influencing vaccine hesitancy in religious individuals may help to inform interventions to increase vaccine uptake in these communities.

#### Limitations and future directions

The current study used a convenience sample of New Zealand residents, and so it is unclear whether our findings will replicate in a larger nationally representative sample, or in other countries. Indeed, because levels of vaccine hesitancy were (on average) low in the current study, and the effect of cognitive flexibility on vaccine hesitancy was small (albeit significant for the personal dimension), replication with a larger and more representative sample is an important next step to verify our findings. Additionally, because our data collection took place during the public roll-out of the COVID-19 vaccine, issues relating to vaccination may have been particularly salient for our participants. As a result, participants’ reported attitudes towards vaccination may have been more (or less) positive than they would have been in the absence of the COVID-19 context. Replicating the current study will help to clarify the extent to which the COVID-19 context may have impacted our findings, and provide additional opportunities to validate the MVHS factor structure.

The Wisconsin Card-Sorting Task (WCST) is a standard neurocognitive measure of cognitive flexibility. However, it is important to recognize that in addition to cognitive flexibility, performance in this task may also reflect other facets of executive functioning or cognition (e.g., response inhibition, working memory; 101). Hence, it is unclear to what extent our results actually reflect the effects of cognitive flexibility per se, versus other cognitive processes, on vaccine hesitancy. To disentangle the unique contribution of cognitive flexibility from other cognitive processes, future work should include additional tasks that measure other components of executive functioning. Additionally, including other measures of cognitive flexibility, such as the Trail Making Test [102] or Alternative Uses Task [103], would also help to clarify the generality of our findings. Taking measures of vaccine behavior (e.g., asking participants whether they have received vaccines in the past) in future studies will also provide insight into whether cognitive flexibility predicts actual vaccine behavior.

As noted previously, our findings are correlational, and so it is unclear whether the relationship between cognitive flexibility and vaccine hesitancy is causal. Hence, based on the present data, we can only tentatively suggest that interventions aiming to reduce vaccine hesitancy should consider the role of cognitive flexibility. To be sure, future work should examine the causal relationship between cognitive flexibility and attitudes towards vaccination. If such a causal relationship exists, this would provide strong evidence that cognitive flexibility indeed underpins attitudes towards vaccination.

## Conclusions

The present findings are the first to show that cognitive flexibility underlies attitudes towards vaccination. Specifically, we found that low cognitive flexibility predicted greater personal, but not external, barriers to vaccination in a convenience sample of New Zealand residents. This has important implications because it suggests that some people – particularly those for whom vaccine hesitancy reflects personal beliefs – may find it more difficult to adapt their attitudes, beliefs, or behaviors even when faced with credible information about the safety, efficacy, or benefits of vaccination. These findings join the growing body of evidence pointing to the role of individual cognitive styles in shaping vaccination attitudes and behaviors, and our findings suggest several fruitful avenues for future research to elucidate the impact of cognitive flexibility on the development, strength, and persistence of vaccine hesitancy.

## Electronic supplementary material

Below is the link to the electronic supplementary material.

Supplementary Material 1

## Data Availability

The datasets generated during the current study are not publicly available due to compliance with data management policies by the institutional ethics committee, but are available from the corresponding author upon request.

## Footnotes

1 The Māori-language name for New Zealand.

2 Extended family group.

3 We also ran correlational analyses using Howard’s (2021) original factor structure. There were weak positive correlations between perseverative responses and scores on the Beliefs, Distrust, Healthy, and Personal Reactions subscales. Correlations between perseverative responses and scores on the other subscales (Access, Cost, Forget, Health Risks, Inconvenience, and Physical Pain) were not significant.

4 Full results of regression analyses with Howard’s (2021) original factor solution are provided in the supplementary materials.

## Abbreviations

CFA: Confirmatory factor analysis
CFI: Comparative fit index
CST: Card–Sorting Task
df: degrees of freedom
EFA: Exploratory factor analysis
IFI: Incremental fit index
MVHS: Multidimensional Vaccine Hesitancy Scale
NZ: Aotearoa New Zealand
NZD: New Zealand dollars
VIF: Variance inflation factor
RMSEA: Root mean squared error of approximation
