# Supplementary material for: Cognitive flexibility predicts attitudes towards vaccination: evidence from a New Zealand sample
Source: BMC Psychol. 2024 Oct 14;12:550. doi: 10.1186/s40359-024-02048-2 (PMC11472431; doi:10.1186/s40359-024-02048-2)
Supplement: Supplementary file 1 — Supplementary Material 1 [file 40359_2024_2048_MOESM1_ESM.docx]

**Supplementary Materials**

**Demographic Questionnaire**

*Note: Participants entered their age as a numeric value, and selected from a list of options for the other questions. Options were drawn from the New Zealand census.*

Please enter your age in years (leave blank if you prefer not to say): ________________

What is your gender?

- Male / Tāne
- Female / Wahine
- Another gender / He ira kē anō
- Prefer not to say

What ethnic group(s) do you belong to? Select all that apply.

- New Zealand European
- Māori
- Samoan
- Cook Island Māori
- Tongan
- Chinese
- Indian
- Middle Eastern
- Latin American / Hispanic
- African
- Other European
- Other Asian
- Other Pacific Peoples
- Other Ethnicity
- Prefer not to say

What is your highest level of education?

- No qualification
- Level 1, 2, or 3 certificate
- Level 5 or 6 diploma
- Bachelor’s degree / Level 7 qualification
- Post-graduate diploma or Honours degree
- Masters degree
- Doctorate degree
- Prefer not to say

What is your religious affiliation?

- No religion
- Buddhism
- Christianity
- Hinduism
- Islam
- Judaism
- Māori religions, beliefs, and philosophies
- Spiritualism and New Age religions
- Other religion
- Prefer not to say

Please select your current NZ region of residence.

- Northland
- Auckland
- Waikato
- Bay of Plenty
- Gisborne
- Hawke’s Bay
- Taranaki
- Manawatū-Whanganui
- Wellington
- Tasman
- Nelson
- Marlborough
- West Coast
- Canterbury
- Otago
- Southland
- Prefer not to say

**Results of Confirmatory Factor Analysis for the MVHS**

**Supplementary Table 1.**

*Confirmatory factor analysis item loadings for the standard Multidimensional Vaccine Hesitancy Scale.*

| Subscale | Item | Factor loading |
| --- | --- | --- |
| Health Risks | Vaccines can cause long-term health issues. | .76 |
|  | Vaccines are unsafe. | .64 |
|  | Vaccines can cause illness. | .79 |
|  | Vaccines can cause certain disorders. | .81 |
| Cost | Vaccines cost too much. | .91 |
|  | I am unable to get vaccines because they cost too much. | .65 |
|  | Vaccines are too expensive. | .98 |
|  | Without health insurance, vaccines cost too much. | .73 |
| Physical Pain | Needles bother me when receiving a vaccine. | .95 |
|  | I worry about needles when getting a vaccine. | .93 |
|  | Getting a vaccine hurts. | .57 |
|  | I have a phobia of needles when receiving a vaccine. | .87 |
| Inconvenience | I am too busy to get a vaccine. | .75 |
|  | Getting a vaccine is too much of a hassle. | .73 |
|  | Getting a vaccine is too much trouble. | .75 |
|  | I do not have the time to get a vaccine. | .78 |
| Personal Reactions | I have allergic reactions to most vaccines. | .83 |
|  | I am a high-risk person for having a negative reaction to vaccines. | .67 |
|  | I am allergic to certain ingredients in vaccines. | .76 |
|  | I have a medical condition that prevents me from getting vaccines. | .57 |
| Access | Vaccines are unavailable where I live. | .65 |
|  | There is nowhere to get a vaccine. | .72 |
|  | It is difficult to get a vaccine where I life. | .85 |
|  | It is difficult to know where to get a vaccine. | .67 |
| Healthy | I do not need vaccines because I rarely get sick. | .79 |
|  | My strong immune system eliminates any need for vaccines. | .82 |
|  | I do not need vaccines because I am a low-risk person. | .89 |
|  | People in my physical condition do not need vaccines. | .90 |
| Forget | Getting vaccines often slips my memory. | .74 |
|  | I just forget about getting vaccines. | .68 |
|  | I just never get around to getting vaccines. | .86 |
|  | I accidentally skip getting vaccines. | .76 |

**Supplementary Table 2.**

*Descriptive statistics for MVHS subscales and results of correlations between each subscale and perseverative response rates.*

|  |  | Full sample (*N* = 601) | |  | Participants with full demographic info (*N* = 520) | |
| --- | --- | --- | --- | --- | --- | --- |
|  | *M* (*SD*) | *r* | *p* |  | *r* | *p* |
| Health Risks | 2.96 (1.34) | .00 | .924 |  | .02 | .305 |
| Cost | 1.99 (1.17) | -.04 | .364 |  | -.03 | .285 |
| Physical Pain | 2.95 (1.67) | .02 | .593 |  | .04 | .179 |
| Inconvenience | 1.97 (1.08) | .00 | .933 |  | .02 | .325 |
| Personal Reactions | 1.56 (0.85) | .11 | .008** |  | .12 | .003** |
| Access | 1.47 (0.69) | .07 | .111 |  | .07 | .050 |
| Healthy | 1.83 (1.12) | .10 | .017* |  | .11 | .006** |
| Forget | 2.41 (1.33) | -.06 | .112 |  | -.05 | .115 |
| Distrust | 2.28 (1.35) | .10 | .014 |  | .12 | .003** |
| Beliefs | 1.51 (0.94) | .14 | < .001 |  | .17 | < .001*** |

* *p* < .05, ** *p* < .01, *** *p* < .001

*Note.* Correlations were conducted using the full sample, and using the sample of participants that provided full demographic information.

**Results of Linear Regressions with MVHS Subscales**

**Supplementary Table 3.**

Linear regressions with perseverative responses and demographic variables as predictors of the Health Risks, Cost, and Physical Pain dimensions from the standard MVHS.

|  | **Health Risks** | | |  | **Cost** | | |  | **Physical Pain** | | |
| --- | --- | --- | --- | --- | --- | --- | --- | --- | --- | --- | --- |
| **Predictor** | **β** | **t** | **p** |  | **β** | **t** | **p** |  | **β** | **t** | **p** |
|  | ***F*(10, 509) = 4.26, *p* < .001***** | | |  | ***F*(10, 509) = 2.06, *p* = .026*** | | |  | ***F*(10, 509) = 2.64, *p* = .004**** | | |
| Perseverative responses | 0.02 | 0.37 | .714 |  | -0.07 | 1.47 | .143 |  | 0.08 | 1.67 | .096 |
| Age | 0.06 | 1.41 | .159 |  | -0.07 | 1.53 | .126 |  | -0.18 | 3.95 | < .001*** |
| Gender (ref: male) | -0.10 | 2.26 | .024* |  | -0.04 | 1.01 | .312 |  | 0.02 | 0.56 | .579 |
| Education level | -0.06 | 1.32 | .189 |  | -0.09 | 1.93 | .054 |  | -0.04 | 0.79 | .431 |
| Ethnicity (ref: European) |  |  |  |  |  |  |  |  |  |  |  |
| *Māori* | 0.08 | 1.84 | .066 |  | 0.02 | 0.51 | .612 |  | 0.01 | 0.17 | .868 |
| *Pacific peoples* | 0.09 | 2.06 | .040* |  | -0.03 | 0.56 | .575 |  | -0.09 | 2.10 | .036* |
| *Asian* | -0.19 | 3.98 | < .001*** |  | 0.06 | 1.18 | .240 |  | -0.05 | 0.99 | .321 |
| *Other* | -0.05 | 1.04 | .298 |  | 0.00 | 0.09 | .930 |  | 0.05 | 1.14 | .256 |
| Religion (ref: no religion) |  |  |  |  |  |  |  |  |  |  |  |
| *Christian* | 0.09 | 1.81 | .072 |  | 0.09 | 1.86 | .063 |  | -0.00 | 0.05 | .964 |
| *Other religion* | 0.06 | 1.38 | .169 |  | 0.13 | 2.85 | .005** |  | 0.01 | 0.26 | .798 |

* *p* < .05, ** *p* < .01, *** *p* < .001

**Supplementary Table 4.**

Linear regressions with perseverative responses and demographic variables as predictors of the Inconvenience, Personal Reactions, and Access dimensions from the standard MVHS.

|  | **Inconvenience** | | |  | **Personal Reactions** | | |  | **Access** | | |
| --- | --- | --- | --- | --- | --- | --- | --- | --- | --- | --- | --- |
| **Predictor** | **β** | **t** | **p** |  | **β** | **t** | **p** |  | **β** | **t** | **p** |
|  | ***F*(10, 509) = 1.98, *p* = .033*** | | |  | ***F*(10, 509) = 1.86, *p* = .048*** | | |  | ***F*(10, 509) = 3.39, *p* < .001***** | | |
| Perseverative responses | 0.01 | 0.30 | .766 |  | 0.08 | 1.81 | .071 |  | 0.03 | 0.57 | .568 |
| Age | -0.14 | 2.96 | .003** |  | 0.05 | 1.05 | .296 |  | -0.08 | 1.80 | .072 |
| Gender (ref: male) | -0.02 | 0.36 | .717 |  | 0.05 | 1.11 | .268 |  | -0.11 | 2.61 | .009** |
| Education level | -0.06 | 1.36 | .173 |  | -0.08 | 1.67 | .095 |  | -0.08 | 1.74 | .083 |
| Ethnicity (ref: European) |  |  |  |  |  |  |  |  |  |  |  |
| *Māori* | -0.01 | 0.25 | .805 |  | 0.05 | 1.15 | .251 |  | 0.05 | 1.20 | .230 |
| *Pacific peoples* | -0.05 | 1.01 | .311 |  | -0.01 | 0.26 | .798 |  | -0.06 | 1.41 | .158 |
| *Asian* | -0.02 | 0.51 | .609 |  | 0.02 | 0.47 | .638 |  | 0.07 | 1.39 | .164 |
| *Other* | -0.07 | 1.63 | .103 |  | -0.04 | 0.86 | .390 |  | -0.02 | -0.54 | .588 |
| Religion (ref: no religion) |  |  |  |  |  |  |  |  |  |  |  |
| *Christian* | 0.09 | 1.94 | .053 |  | 0.08 | 1.62 | .106 |  | 0.14 | 2.89 | .004** |
| *Other religion* | 0.07 | 1.48 | .141 |  | 0.07 | 1.59 | .112 |  | 0.08 | 1.72 | .086 |

* *p* < .05, ** *p* < .01, *** *p* < .001

**Supplementary Table 5.**

Linear regressions with perseverative responses and demographic variables as predictors of the Healthy and Forget dimensions of the standard MVHS.

|  | **Healthy** | | |  | **Forget** | | |
| --- | --- | --- | --- | --- | --- | --- | --- |
| **Predictor** | **β** | **t** | **p** |  | **β** | **t** | **p** |
|  | ***F*(10, 509) = 3.69, *p* < .001***** | | |  | ***F*(10, 509) = 5.35, *p* < .001***** | | |
| Perseverative responses | 0.08 | 1.69 | .091 |  | -0.03 | 0.74 | .462 |
| Age | -0.07 | 1.37 | .171 |  | -0.22 | 5.01 | < 001*** |
| Gender (ref: male) | -0.09 | 2.11 | .035* |  | -0.03 | 0.63 | .530 |
| Education level | -0.05 | 1.10 | .272 |  | -0.13 | 3.06 | .002** |
| Ethnicity (ref: European) |  |  |  |  |  |  |  |
| *Māori* | 0.01 | 0.23 | .822 |  | 0.02 | 0.55 | .586 |
| *Pacific peoples* | -0.01 | 0.29 | .769 |  | -0.05 | 1.15 | .252 |
| *Asian* | -0.10 | 2.14 | .033* |  | -0.10 | 2.09 | .038* |
| *Other* | -0.06 | 1.34 | .180 |  | -0.08 | 1.76 | .079 |
| Religion (ref: no religion) |  |  |  |  |  |  |  |
| *Christian* | 0.21 | 4.43 | < .001*** |  | 0.08 | 1.75 | .081 |
| *Other religion* | 0.10 | 2.19 | .029* |  | 0.07 | 1.64 | .101 |

* *p* < .05, ** *p* < .01, *** *p* < .001

**Supplementary Table 6.**

Linear regressions with perseverative responses and demographic variables as predictors of Beliefs and Distrust from the extended MVHS.

|  | **Beliefs** | | |  | **Distrust** | | |
| --- | --- | --- | --- | --- | --- | --- | --- |
| **Predictor** | **β** | **t** | **p** |  | **β** | **t** | **p** |
|  | ***F*(10, 509) = 5.16, *p* < .001** | | |  | ***F*(10, 509) = 3.63, *p* < .001***** | | |
| Perseverative responses | 0.10 | 2.20 | .028* |  | 0.08 | 1.78 | .075 |
| Age | -0.01 | 0.15 | .884 |  | -0.00 | 0.05 | .960 |
| Gender (ref: male) | -0.06 | 1.39 | .165 |  | -0.08 | 1.73 | .084 |
| Education level | -0.08 | 1.63 | .105 |  | -0.06 | 1.29 | .198 |
| Ethnicity (ref: European) |  |  |  |  |  |  |  |
| *Māori* | 0.02 | 0.46 | .644 |  | 0.01 | 0.18 | .856 |
| *Pacific peoples* | 0.04 | 0.98 | .325 |  | 0.03 | 0.70 | .485 |
| *Asian* | -0.03 | 0.58 | .560 |  | -0.09 | 2.01 | .045* |
| *Other* | -0.04 | 0.92 | .359 |  | -0.07 | 1.56 | .120 |
| Religion (ref: no religion) |  |  |  |  |  |  |  |
| *Christian* | 0.24 | 5.05 | < .001*** |  | 0.19 | 3.96 | < .001*** |
| *Other religion* | 0.12 | 2.73 | .007** |  | 0.12 | 2.61 | .009** |

* *p* < .05, ** *p* < .01, *** *p* < .001
